# Effect of pancrelipase in preventing pancreatic dysfunction after pancreaticoduodenectomy

**DOI:** 10.20407/fmj.2018-016

**Published:** 2019-04-17

**Authors:** Yukio Asano, Akihiko Horiguchi, Masahiro Ito, Satoshi Arakawa, Shinpei Furuta, Masahiro Shimura, Chihiro Hayashi, Kenshiro Kamio, Toki Kawai, Hironobu Yasuoka, Takahiko Higashiguchi

**Affiliations:** Department of Gastroenterological Surgery, Fujita Health University Bantane Hospital, Nagoya, Aichi, Japan

**Keywords:** Pancrelipase, Pancreaticoduodenectomy, Nutrition assessment, Pancreatectomy

## Abstract

**Objectives:**

Patients who have undergone pancreaticoduodenectomy (PD) may experience a long-term decrease in quality of life because of postoperative pancreatic dysfunction (such as digestive and absorption disorders) and fatty liver as a result of combined resection of the duodenum, gallbladder, and bile duct. The present study investigated the usefulness of pancrelipase for the prevention of pancreatic dysfunction after PD.

**Methods:**

The data from 73 patients who underwent PD in a single institution were analyzed. Patients who underwent PD during 2007–2011 were administered the low-titer pancreatic enzyme preparations berizym^®^ and pancreatin^®^ (first period group), while patients who underwent PD during 2012–2017 were administered the high-titer pancreatic enzyme preparation pancrelipase (second period group). The following measures of the nutrition status were examined before and after PD: serum albumin concentration, total lymphocyte count, serum total cholesterol concentration, body mass index, controlling nutrition status (CONUT) index, Onodera’s prognostic nutrition index (PNI), and liver computed tomography values.

**Results:**

The second period group had significantly higher serum albumin concentrations at 3 and 6 months postoperatively, serum total cholesterol concentrations at 1 month postoperatively, and Onodera’s PNI values at 3 and 6 months postoperatively than the first period group. The CONUT index values at 6 months after PD were significantly lower in the second period group than in the first period group.

**Conclusions:**

Pancrelipase is useful in improving the nutrition status and preventing fatty liver after PD.

## Introduction

Pancreaticoduodenectomy (PD) is the standard operative procedure most commonly used to remove lesions located in the pancreatic head. In some cases, PD results in deteriorated quality of life because of postoperative pancreatic dysfunction (such as digestive and absorption disorders) and fatty liver due to combined resection of the duodenum, gallbladder, and bile duct.^1,2^ Studies have reported that pancreatic dysfunction typified by chronic pancreatitis is effectively managed via the administration of a newly available high-titer pancreatic enzyme preparation called pancrelipase.^3,4^ Therefore, we investigated the usefulness of pancrelipase in preventing pancreatic dysfunction after PD.

## Materials and Methods

The present study included 73 patients who underwent PD at the Department of Gastroenterological Surgery of Fujita Health University between 2007 and 2017. Patients who underwent PD between 2007 and 2011 were administered the low-titer pancreatic enzyme preparations berizym^®^ and pancreatin^®^ (first period group), while patients who underwent PD between 2012 and 2017 were administered the high-titer pancreatic enzyme preparation pancrelipase (second period group). The two groups were compared regarding background characteristics, age, sex, body mass index, condition for which PD was performed, extent of lymph node dissection, presence or absence of total nerve dissection, reconstruction method, preoperative examination data (serum albumin concentration, total lymphocyte count, serum total cholesterol concentration, Onodera’s prognostic nutrition index (PNI),^5^ controlling nutrition status (CONUT) index,^6^ and liver computed tomography (CT) values), and duration of postoperative hospitalization. Additionally, the nutrition status was measured via assessments of the serum albumin concentrations, total lymphocyte counts, serum total cholesterol concentrations, Onodera’s PNI values, CONUT index values, and liver CT values before PD and at 1, 3, 6, and 12 months after PD.

The present study was approved by the Medical Research Ethics Committee of Fujita Health University (approval no. HM17-372). Informed consent was not required due to the retrospective nature of the study.

## Statistical Analysis

Data were analyzed using IBM SPSS statistics 22.0 (IBM Japan, Inc., Tokyo, Japan) Student’s t-tests and chi-squared tests were used to assess the significance of intergroup differences. P<0.05 was considered statistically significant.

## Results

Between 2007 and 2017, 128 patients underwent pancreatectomy, while 73 patients underwent PD in our department. Of the patients who underwent PD, 34 were in the first period group, while 39 were in the second period group. The only preoperative background characteristics that significantly differed between the first period group and the second period group were the condition for which PD was performed and the liver CT value (Table 1). In all patients, the reconstruction method was subtotal stomach-preserving pancreaticoduodenectomy-II, in which the anastomoses of the jejunum are performed in the order of pancreas, bile duct, and stomach from the oral side.

**Figure 1:**
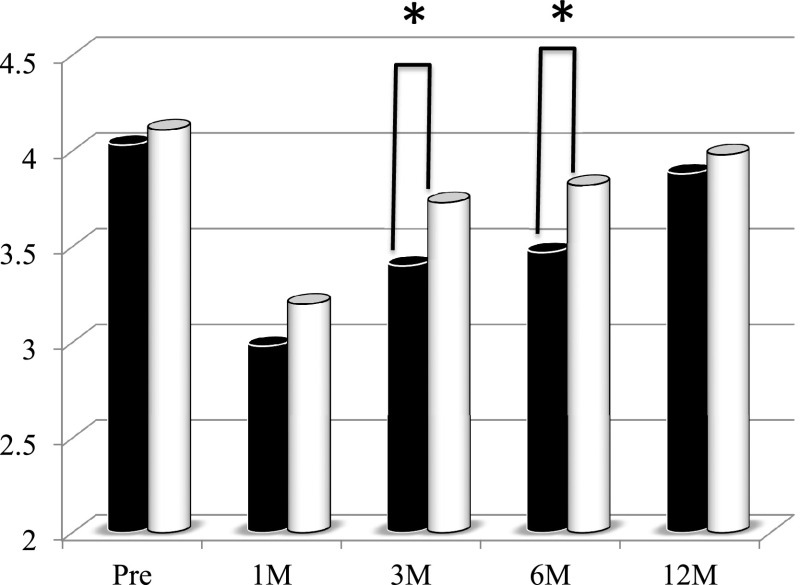
Changes in serum albumin concentrations in patients who underwent pancreaticoduodenectomy in the first period (with low-titer digestive enzyme preparations) versus the second period (with high-titer pancrelipase) The serum albumin concentrations at 3 and 6 months postoperatively were significantly higher in the second period group (white bar) than in the first period group (black bar). *p<0.05

**Figure 2:**
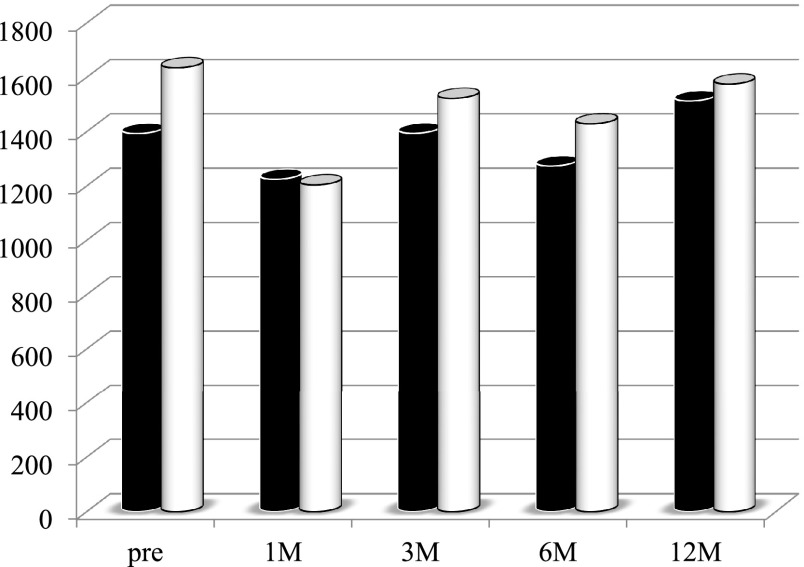
Changes in total lymphocytes counts in patients who underwent pancreaticoduodenectomy in the first period (with low-titer digestive enzyme preparations) versus the second period (with high-titer pancrelipase) There was no significant intergroup difference in the total lymphocyte count.

**Figure 3:**
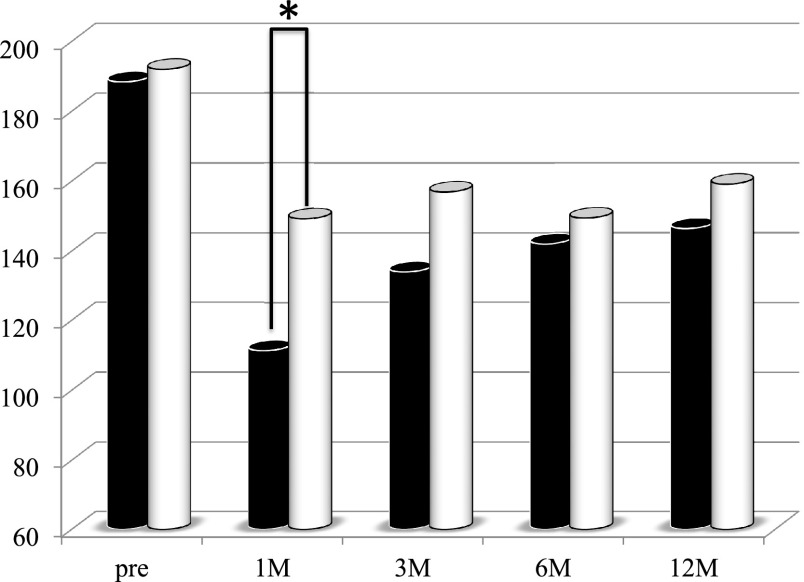
Changes in serum cholesterol in patients who underwent pancreaticoduodenectomy in the first period (with low-titer digestive enzyme preparations) versus the second period (with high-titer pancrelipase) The serum total cholesterol concentrations at 1 month postoperatively were significantly higher in the second period group (white bar) than in the first period group (black bar). *p<0.05

**Figure 4:**
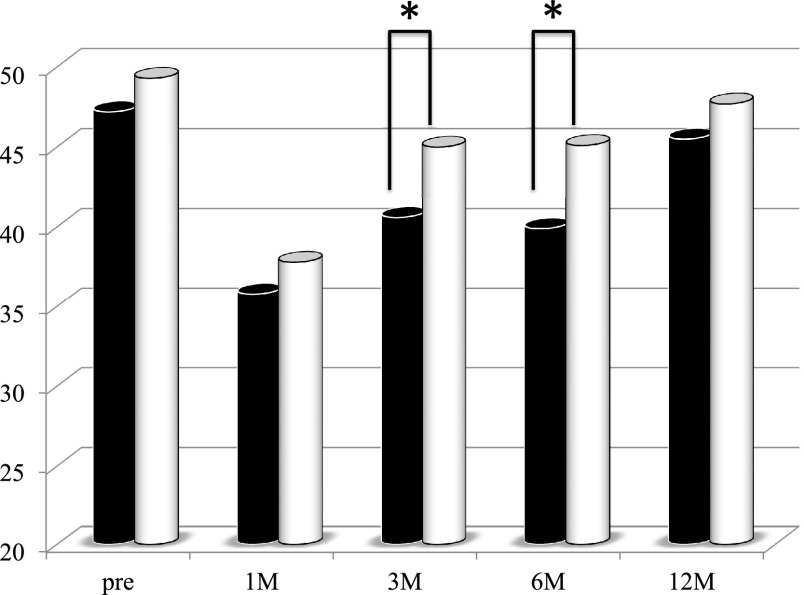
Changes in the Onodera’s prognostic nutrition index (PNI) values of the patients who underwent pancreaticoduodenectomy in the first period (with low-titer digestive enzyme preparations) versus the second period (with high-titer pancrelipase) The Onodera’s PNI values at 3 and 6 months postoperatively were significantly higher in the second period group (white bar) than in the first period group (black bar). *p<0.05

**Figure 5:**
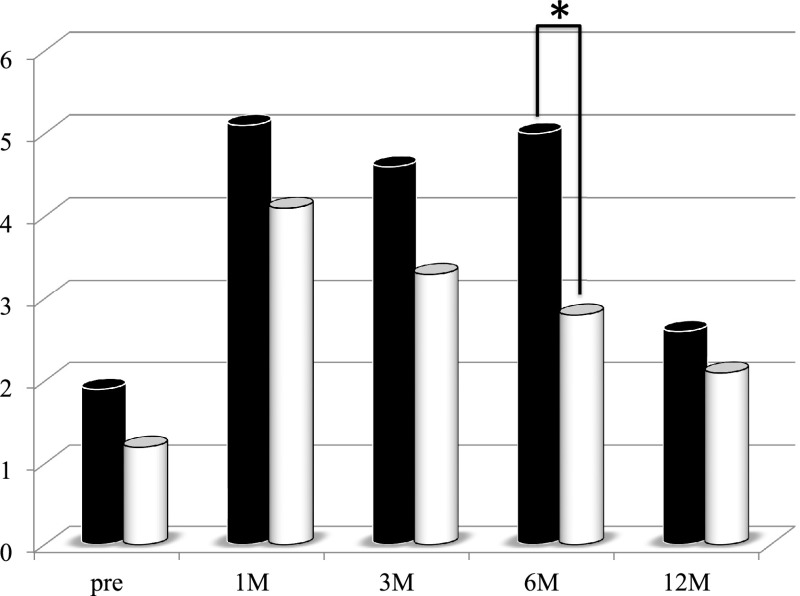
Changes in the controlling nutrition status (CONUT) index values of the patients who underwent pancreaticoduodenectomy in the first period (with low-titer digestive enzyme preparations) versus the second period (with high-titer pancrelipase) CONUT index values at 6 months postoperatively were significantly lower in the second period group (white bar) than in the first period group (black bar). *p<0.05

**Figure 6:**
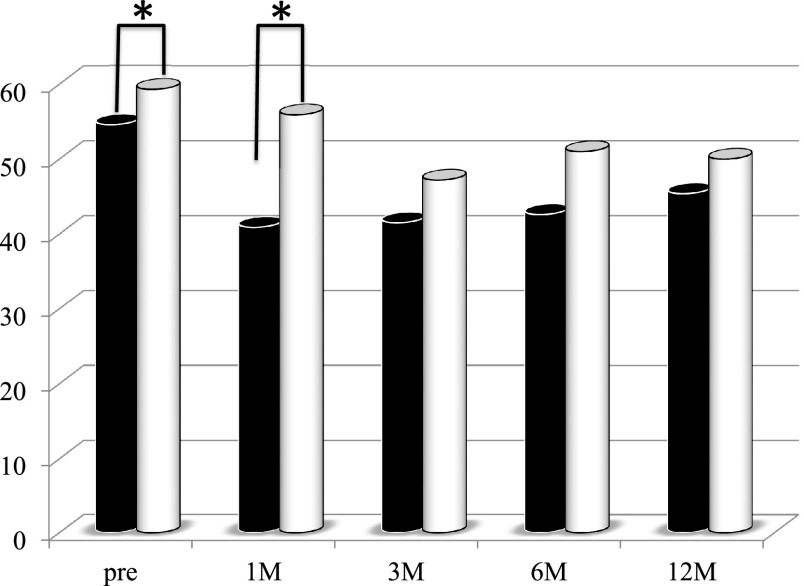
Changes in the liver computed tomography values of the patients who underwent pancreaticoduodenectomy in the first period (with low-titer digestive enzyme preparations) versus the second period (with high-titer pancrelipase) The liver computed tomography values before surgery and at 1 month postoperatively were significantly lower in the first period group (black bar) than in the second period group (white bar). *p<0.05

The serum albumin concentrations at 3 and 6 months after PD were significantly higher in the second period group than in the first period group (Figure 1). There was no significant difference in the total lymphocyte count between the groups (Figure 2). The serum total cholesterol concentration at 1 month after PD was significantly higher in the second period group than in the first period group (Figure 3). The Onodera’s PNI values at 3 and 6 months after PD were significantly higher in the second period group than in the first period group (Figure 4). The CONUT index value at 6 months after PD was significantly lower in the second period group than in the first period group (Figure 5). The liver CT values before and at 1 month after PD were significantly lower in the first period group than in the second period group (Figure 6).

## Discussion

The safety of PD has been increased by improvements in preoperative diagnosis, surgical procedure, and postoperative management, resulting in fewer perioperative complications such as pancreatic fistula.^7^ Additionally, the postoperative outcome of PD for pancreatic carcinoma has been improved by advances in adjuvant chemotherapy, with some patients achieving long-term survival.^8^ However, long-term survivors after PD can experience long-term complications because of pancreatic dysfunction.

The digestion and absorption of fat are closely related to pancreatic function. Pancreatic juice contains lipase, which hydrolyzes orally ingested fat to produce free fatty acids that are subsequently micellized by bile acid. The micellized fatty acids are absorbed via the epithelial cells of the small intestine, then mostly migrate to the portal vein system, and are metabolized in the liver to rapidly produce a source of energy. A minority of these micellized fatty acids migrate to the thoracic duct via lymph channels. After PD, the concentrations of various digestive enzymes in the pancreatic juice are relatively reduced, and the pancreatic juice secretion decreases because of the reduced secretion of cholecystokinin and secretin resulting from resection of the duodenum. Steatorrhea reportedly occurs when the amylase secretion level is ≤15% of that in healthy subjects,^9^ or when the lipase secretion level decreases to ≤10% of that in healthy subjects.^10^

It is important to consider the possibility of digestive and absorption disorders when managing patients after PD. In most cases of pancreatic ductal adenocarcinoma, pancreatic fibrosis is highly advanced preoperatively because of the concomitant pancreatitis that develops because of the occlusion of the main pancreatic duct. Additionally, malnutrition is worsened because of nervous diarrhea, as the nerve plexuses around the superior mesenteric artery are often completely removed to achieve a radical cure. This disrupts the digestion and absorption of fat, resulting in malnutrition. Furthermore, there is an increase in the conversion of carbohydrates to fat in the liver, causing the development of fatty liver.

Treatment options for digestive and absorption disorders include digestive enzyme replacement therapy. The development of digestive enzyme preparations started with the formulation of the world’s first therapeutic preparation (taka-diastase) in 1899.^11^ Diastase and biodiastase were developed approximately 50 years later, while pancreatin derived from porcine pancreas was developed in 1953 and remained in mainstream use until recently. However, compared with conventional pancreatin, the enzymatic activities of lipase, amylase, and protease are eight, six, and seven times higher, respectively. Thus, highly extracted/purified pancreatin from porcine pancreas was used to create pancrelipase, which is a high-titer preparation with an enteric coating to prevent inactivation in the stomach.^12^ LipaCreon^®^ is a high-titer pancrelipase formulation that has been covered by the national insurance system in Japan since 2011 for pancreatic secretion insufficiency represented by chronic pancreatitis.^3,4^ Kuroda et al.^13^ reported that 20,000–50,000 units of fat digestion activity per meal are required to be supplemented in a patient with pancreatic dysfunction. As the fat digestibility per gram of pancrelipase is apparently greater than other enzyme preparations, pancrelipase is the current mainstream therapeutic medication for patients with pancreatic dysfunction.^9,13^

In the present study, the nutritional status after PD showed a tendency to recover almost to the preoperative value from 6 months to 1 year postoperatively. However, the improvement in the nutritional status took longer in the first period group (patients who received conventional low-titer digestive enzyme preparations) than in the second period group (patients who received the high-titer digestive enzyme preparation pancrelipase).

There were significant differences in the background diseases in the first period group versus the second period group. However, the main reason that the characteristics of the background diseases in each group is important is because pancreatic ductal adenocarcinoma accompanied by concomitant pancreatitis, lymph node dissection, and nerve plexus dissection causes postoperative malabsorption. Thus, as the incidences of pancreatic ductal adenocarcinoma in both groups were similar, the impact of the intergroup difference in the background diseases was likely to be minimal.

As the preoperative liver CT values were also significantly different between the two groups, this parameter could not be used to evaluate postoperative intergroup differences in nutritional status. However, there were no preoperative intergroup differences in the serum concentrations of albumin and total cholesterol, and the Onodera’s PNI and CONUT index values. The postoperative evaluations of these indicators showed that the nutritional status was significantly better in the second period group (who received the high-titer digestive enzyme preparation pancrelipase) than in the first period group (who received low-titer digestive enzyme preparations).

## Conclusion

High-titer pancrelipase as part of nutrition management is useful for improving the nutritional status and preventing fatty liver after PD.

## Figures and Tables

**Table1:** Preoperative patient background characteristics

		First period groups	Second period groups	p-values
Age		65.9	70.0	0.144
Gender (M:F)		23:11	23:16	0.451
BMI (kg/m^2^）		28.4	21.1	0.256
Disease	PDAC	14	17	0.018*
	BD Ca	11	3	
	Pap. Ca	5	3	
	IPMC	1	6	
	IPMA	3	6	
	Other	0	4	
Lymph node dissection (D1:D2)		4:30	14:25	0.451
Plexus dissection (+:–)		14:20	17:22	0.838
Serum albumin levels		4.0	4.1	0.509
Total lymphocyte counts		1392.8	1591.5	0.148
Serum total cholesterol levels		188.4	194.5	0.450
Onodera’s PNI		47.2	49.0	0.143
CONUT score		1.9	1.2	0.051
Liver CT values		54.6	59.2	0.003*
Hospital Stay		54.9	68.4	0.602

*: significantly different BMI: body mass index, PDAC: pancreatic ductal adenocarcinoma, BD Ca: distal carcinoma of bile duct , Pap. Ca: carcinoma of papilla of Vater, IPMC: Intra-ductal Papillary mucinous carcinoma, IPMA: Intra-ductal Papillary mucinous adenoma
